# Immunpathogenese des systemischen Lupus erythematodes

**DOI:** 10.1007/s00393-022-01214-4

**Published:** 2022-05-13

**Authors:** Martin Aringer, Stephanie Finzel, Reinhard E. Voll

**Affiliations:** 1 Rheumatologie, Medizinische Klinik III und UniversitätsCentrum für Autoimmun- und Rheumatische Erkrankungen (UCARE), Universitätsklinikum und Medizinische Fakultät Carl Gustav Carus, TU Dresden, Fetscherstr. 74, 01307 Dresden, Deutschland; 2 Klinik für Rheumatologie und Klinische Immunologie & Centrum für chronische Immundefizienz, Universitätsklinikum Freiburg, Freiburg, Deutschland

**Keywords:** Antikörper, Immunkomplexe, Plasmazellen, BLyS/BAFF, Interferon, Antibodies, Immune complexes, Plasma cells, BLyS/BAFF, Interferon

## Abstract

Das Verständnis der Immunpathogenese des systemischen Lupus erythematodes (SLE) hilft, das komplexe Krankheitsgeschehen zu verstehen und neue Therapiestrategien zu entwickeln. Die Krankheitsmanifestationen des SLE sind im Wesentlichen Folge von Autoantikörpern, Immunkomplexen und Zytokinen. Insbesondere die Neigung zu unterschiedlichen Autoantikörpern macht das Wesen der Erkrankung aus; die genauen Spezifitäten der Autoantikörper führen zu ganz unterschiedlichen Organmanifestationen. Diese Übersichtsarbeit stellt den klinisch relevanten Stand des Wissens zur SLE-Pathogenese dar – mit dem Ziel, ein für den klinischen Einsatz nützliches Modell zu etablieren, das auch hilft, die neuen Therapieansätze einzuordnen.

## Abstract (en)

Insights into the immunopathogenesis of systemic lupus erythematosus (SLE) help to understand the complex disease patterns and to develop new treatment strategies. The disease manifestations essentially result from autoantibodies, immune complexes and cytokines. Particularly the propensity towards developing various autoantibodies is central to the disease itself; autoantibody specificities lead to highly variable organ manifestations. This review article delineates the clinically relevant state of knowledge on SLE pathogenesis, with the goal to establish a model useful for clinical practice, which also helps to classify the novel therapeutic approaches.

Der SLE ist eine systemische Autoimmunkrankheit mit noch unvollständig verstandener Ätiologie und Immunpathogenese. Neben einer polygenen genetischen Komponente – es sind inzwischen weit über 100 Risiko-Loci bekannt – spielen Umweltfaktoren wie UV-Exposition, virale Infektionen (EBV), Rauchen und wahrscheinlich Vitamin-D-Mangel [1, 2] eine Rolle.

Ohne Verständnis der immunologischen Grundlagen der Pathogenese ist der SLE schwer zu fassen. Immunologische Grundlagen sind daher noch entscheidender als bei vielen anderen systemischen Autoimmunerkrankungen und entzündlich rheumatischen Krankheitsbildern. Nur über die Klammer der verschiedenen Autoantikörper bleibt der SLE in seiner Vielfalt an Krankheitsmanifestationen [3–6] durchschaubar. Mit einem relativ einfachen Bild der Immunologie (Abb. 1) steht uns ein Schlüssel zu einem Verständnis des SLE zur Verfügung, das direkte Konsequenzen für die Diagnostik und das Management der Erkrankung hat.

**Figure 1:**
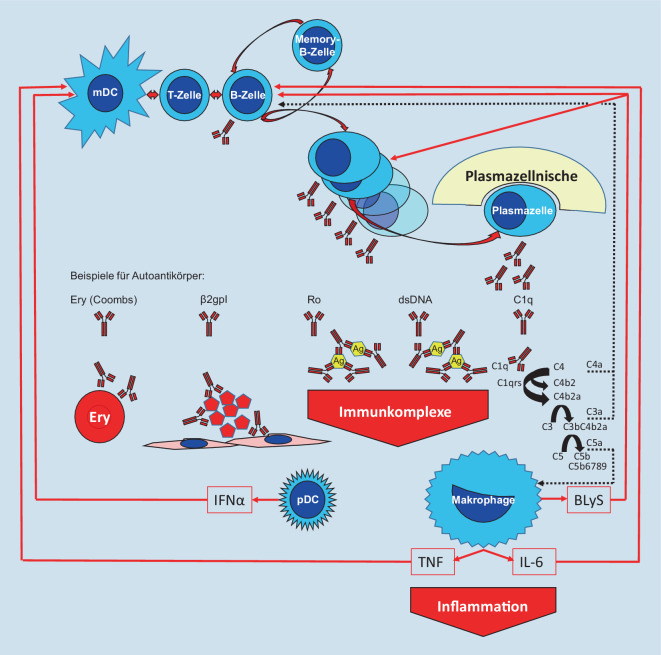

Autoantikörper sind der zentrale Angelpunkt der Erkrankung [7]. Bevor wir die Faktoren analysieren werden, die genetisch oder als Umwelteinflüsse den Ausbruch der Erkrankung begünstigen, lohnt es sich, den Fokus auf die Autoantikörperwelt des SLE zu legen. Wenn man versucht, diese Autoantikörper zu gruppieren, fällt einem auf, dass sich ein relevanter Teil gegen Ribonukleinsäuren richtet – oder gegen Proteine, die Ribonukleinsäuren binden [8, 9]. Dazu gehören einerseits die charakteristischen Antikörper gegen doppelsträngige Desoxyribonukleinsäure (dsDNA) und gegen Histone sowie ganze Nukleosomen und andererseits Antikörper gegen RNA-bindende Proteine, insbesondere Sm, U1RNP, Ro (SS-A) und La (SS-B), aber auch hnRNP A2 (RA33) und Phospholipide (Cardiolipin) bzw. Phospholipid-bindende Proteine wie Beta-2-Gykoprotein‑I (Tab. 1). Typischerweise haben alle diese Autoantikörper eine elektrische Ladung, die zum Teil wieder für die Ablagerung der daraus entstehenden Immunkomplexe an Basalmembranen wichtig ist. Die spezifischen SLE-Autoantikörper gehören in diese Kategorie.

**Table 1:** 

**Antikörper mit elektrischer Ladung**	
*Antigen*	*Testsystem*
dsDNA^a^	Farr, CLIFT, CIA, ELISA
Histone	ELISA
Nukleosomen	ELISA
Sm^a^	LIA, Immundiffusion, ELISA
U1RNP	LIA, Immundiffusion, ELISA
Ro	LIA, Immundiffusion, ELISA
La	LIA, Immundiffusion, ELISA
RA33 (hnRNP A2)	ELISA
Anti-Cardiolipin^a^	ELISA
Anti-β2-gp I^a^	ELISA
**Antikörper ohne elektrische Ladung**	
*Antigen*	*Testsystem*
Erythrozyten	Coombs-Test
Thrombozyten	Indirekt (Blutbild), ELISA
Leukozyten	Indirekt (Blutbild)
Lymphozyten	Indirekt (Blutbild)
C1q	ELISA
Ribosomal P	ELISA

*CLIFT* Crithidia luciliae Immunfluoreszenztest, *CIA* chemiluminscence immunoassay, *ELISA* enzyme linked immunsorbent assay, *LIA* line immune assay

^a^Für die SLE-Klassifikation relevante Autoantikörpertests (SLE-spezifische Antikörper, Antiphospholipidantikörper)

Die andere Gruppe von Autoantikörpern ist hingegen zumindest nicht offensichtlich bzw. regelhaft elektrisch geladen. Dazu gehören unter anderem die Autoantikörper gegen diverse Blutzellen, die entsprechend zu Coombs-positiver autoimmunhämolytischer Anämie, Thrombopenie, Leukopenie oder Lymphopenie führen können, aber auch Antikörper gegen die Komplementkomponente C1q, ribosomale P‑Proteine und viele Autoantikörper geringer Spezifität, wie z. B. Schilddrüsenautoantikörper und Autoantikörper gegen Antigene fraglicher Relevanz [10, 11].

## Wege zur Autoantikörperbildung

Damit Autoantikörper entstehen können, müssen mindestens 2 Voraussetzungen gegeben sein: Einerseits muss das Autoantigen irgendwann dem Immunsystem zugänglich sein. Andererseits müssen Kontrollmechanismen, welche die Proliferation autoimmuner B‑Zellen verhindern, außer Kraft gesetzt werden. Dazu kommt noch eine (naturgemäß genetisch bedingte) HLA-Konfiguration, die eine Präsentation bestimmter Antigenpeptide ermöglicht.

Die SLE-typischen Autoantikörper haben Immunglobulinklassenwechsel und Affinitätsreifung durchgemacht. Im Rahmen der Affinitätsreifung kommt es zu zufälligen Mutationen im Bereich der Antigen-bindenden Domäne der Antikörpermoleküle. In der Folge können nur die B‑Zellen überleben und klonal expandieren, deren B‑Zell-Rezeptoren (membranständige Antikörper) durch die Mutationen eine höhere Affinität zum Antigen erworben haben. Die Gensequenz der Immunglobuline mutiert in den variablen Regionen besonders stark und entspricht somit nicht mehr der Keimbahnsequenz, die bei naiven, noch nicht aktivierten B‑Zellen, gefunden wird.

Im Gegensatz zu solchen „Germline“-Antikörpern finden sich bei SLE-assoziierten Autoantikörpern typischerweise Mutationen, die zum Austausch von Aminosäuren führen. So besitzen Anti-dsDNA-Antikörper oft positiv geladene Aminosäuren, v. a. Arginin, in den Antigen-bindenden Domänen. Dadurch steigt die Affinität zur negativ geladenen DNA. Da sowohl Immunglobulinklassenwechsel als auch Affinitätsreifung weitgehend T‑Zell-abhängige Prozesse darstellen, hatten die autoreaktiven B‑Zellen während ihrer Reifung T‑Zell-Hilfe, also Unterstützung durch Antigen-spezifische T‑Helferzellen [12, 13], insbesondere follikuläre T‑Helferzellen (T_FH_). Auch hier sollten Sicherheitsmechanismen greifen, die normalerweise zur (zentralen oder peripheren) Toleranz führen, aber beim SLE offensichtlich unzureichend funktionieren.

## Zugang zu Autoantigenen

Viele der SLE-Autoantigene sind normalerweise tief im Inneren der Zellen verborgen, klassischerweise im Zellkern (antinukleäre Antikörper [ANA]). Zugänglich werden sie typischerweise infolge des Zelltods, der auf unterschiedliche Art und Weise erfolgen kann.

Physiologisch sterben Zellen durch Apoptose, gleichsam aus Altersschwäche oder weil sie nicht mehr benötigt werden, aber z. B. auch durch exogene Schädigung wie UV-Bestrahlung. Apoptose ist ein energieabhängiger Prozess, der durch endogene Funktionsstörungen (z. B. der Mitochondrien, DNA-Schädigung) oder exogene Selbstmordbefehle (z. B. durch Bindung von Fas-Ligand an Fas; Fehlen von Wachstums- oder Überlebensfaktoren wie Interleukin-2 (IL‑2) für aktivierte T‑Zellen) ausgelöst wird und bei dem die Zelle „gefährliche“ Antigene gleichsam einpackt und mit „immunregulierenden“ Signalen und „Friss-mich“-Signalen kombiniert [14, 15]. Obwohl diese Autoantigene so relativ gut zugänglich sind, würden sie noch nicht zu einer Immunreaktion führen. Die apoptotischen Zellreste werden dank der „Friss-mich“-Signale normalerweise sehr effizient durch Fresszellen aufgenommen, und es kommt nicht zu einer Autoimmunreaktion. Das ändert sich, wenn die Phagozytose durch spezialisierte Makrophagen zu lange dauert. Dann ähneln die apoptotischen Zellreste immer mehr denen von Zellen, die durch Nekrose, also „gewaltsam“ ums Leben gekommen sind. Nekrotische Zellen setzen Alarmine frei, um das Immunsystem zu aktivieren; schließlich ist die Ursache für Nekrose häufig eine Infektion oder ein physikalisches Trauma mit Störung der Epithelbarriere und sekundärer Infektion oder mitunter auch ein Malignom.

Beim SLE ist diese verzögerte Aufnahme apoptotischen Materials gut gezeigt. Vermutlich liegt sie auch der UV-Empfindlichkeit im Sinne einer Auslösung von Schüben zugrunde, weil bei jeder UV-Exposition Hautzellen apoptotisch werden und beseitigt werden müssen [16]. Ist die Phagozytose der apoptotischen Zellen beeinträchtigt, werden diese sekundär nekrotisch und setzen unter anderem Nukleosomen frei, die mit proinflammatorischen Alarminen wie HMGB1 assoziiert sind. Diese Protein-DNA-Komplexe können dendritische Zellen effizient aktivieren, worauf diese z. B. Histonpeptide präsentieren und gleichzeitig kostimulatorische Signale exprimieren [17]. Ziemlich sicher gibt es unterschiedliche Mechanismen, die die sofortige Phagozytose apoptotischer Zellen beeinträchtigen. Dazu gehören hereditäre Defekte früher Komplementkomponenten wie C1q, C2 und C4, da Komplement auch an der Beseitigung spät-apoptotischer Zellen beteiligt ist [18]; auch Antikörper gegen Komplementproteine werden beim SLE gefunden und können Funktionsstörungen des Komplementwegs vermitteln. Weitere Mechanismen sind nicht ganz aufgeklärt; sie scheinen aber zum Teil mit der SLE-Aktivität (Immunkomplexe) zusammenzuhängen, zum Teil genetisch determiniert [19] zu sein (Tab. 2).

**Table 2:** 

Gen	Funktion des Proteins	Angenommene Mechanismen
*C1q*	Komplementkomponenten (frühe Komponenten des klassischen Aktivierungswegs)	0: Defekte Abräumung apoptotischer Zellen
*C1r/C1s*		
*C2*		
*C4*		
*DNASE1*	DNA-Endonukleasen	0: Verminderte Degradation von NETs oder anderweitig freigesetzten Chromatins; damit Anreicherung von DNA-assoziierten Autoantigenen sowie vermehrte Interferonantwort auf vermehrte intra- und extrazelluläre DNA-Reste
*DNASE2*		
*DNASE1L3*		
*TREX1*		
*SAMHD1*	Phosphohydrolase	0: Vermehrte Interferonantwort, Anfälligkeit gegen Retroviren
*RNASEH2A*	Ribonukleasen	0: Vermehrte Interferonantwort auf nicht abgebaute RNA
*RNASEH2B*		
*RNASEH2C*		
*ADAR1*	RNA-spezifische Adenosin-Deaminase	
*IFIH1*	dsRNA-Sensor (Prot: MDA5)	+: Vermehrte Interferonantwort durch hyperaktive Rezeptoren
*TMEM173*	Sensor autologe DNA (Prot: SAVI)	
*OTUD1*	Deubiquitinase	0: Vermehrte Interferonantwort durch fehlende Inaktivierung von IRF3
*ACP5*	Tartrat-resistente alkalische Phosphatase (Prot: TRAP)	0: Vermehrte Interferonantwort über TLR9 durch fehlenden Abbau von Osteopontin
*PRKCD*	Proteinkinase Cδ	0: Verminderte Apoptose autoreaktiver B‑Zellen
*FAS/FASL*	Apoptose-Rezeptor/Ligand	0: Verminderte Apoptose von Lymphozyten
*TNFSF13B*	B‑Zell-Zytokin (Prot: BLyS/BAFF)	+: Besseres Überleben autoreaktiver B‑Zellen

*0* Ausfall (oder deutliche Funktionsschwächung), *+* Vermehrte Produktion oder erhöhte Aktivität, *Prot* von Gen kodiertes Protein

Auch bei 2 weiteren Formen des Zelltods werden SLE-Autoantigene dem Immunsystem zugänglich. Einerseits passiert das bei der Nekroptose, einem inflammatorischen Typ des Zelltods, bei dem durch Selbstmordbefehle eine Nekrose ausgelöst wird, mit den oben skizzierten Konsequenzen [14]. Andererseits werden auch bei der NETose Autoantigene zugänglich [20, 21]. NETose ist ein aggressives Selbstmordprogramm neutrophiler und eosinophiler Granulozyten. Dabei werden aus dem Zellinneren Chromatinfragmente, also DNA-Histon-Komplexe aktiv ausgestoßen, um insbesondere bakterielle Krankheitserreger mit klebrigen, netzartigen Strukturen aus Ribonukleinsäure aus dem Neutrophileninneren zu bedecken. In diesen „neutrophilic extracellular traps“ (NETs) befinden sich keimtötende Proteine, wobei insbesondere die kationischen Histone bakterizid wirken. Damit finden sich in den NETs typische Lupusautoantigene wie dsDNA, Histone und HMGB1. Wenn Bakterien wie Staphylokokken eine große Menge neutrophiler Granulozyten ins Gewebe rekrutieren und bei diesen NETose induzieren, entsteht Eiter. Normalerweise werden NETs aber durch DNasen (z. B. DNase I und DNase I like 3) im Blut und Gewebe sofort degradiert und beseitigt. Die Fähigkeit zur Degradation von NETs ist bei einer Subgruppe von SLE-Patienten drastisch beeinträchtigt und dann mit dem Auftreten einer Lupusnephritis assoziiert. Ursache für die verminderte Fähigkeit zur NET-Degradation von Lupusseren ist entweder die Maskierung der NETs durch Anti-dsDNA-Antikörper oder aber die verminderte Aktivität NETs-degradierender DNasen im Serum. Interessanterweise lassen sich NETs z. B. in den entzündeten Nieren bei der Lupusnephritis nachweisen [22]. Da einerseits bei SLE-Patienten durch die hohe Zahl an Low-density-Granulozyten vermehrt Apoptose auftritt, andererseits die NETs-Degradation beeinträchtigt ist, kommen nukleäre Autoantigene in Form von NETs in proinflammatorischem Kontext (z. B. in den NETs gefangenen Bakterien) vermehrt in Kontakt mit antigenpräsentierenden Zellen des angeborenen Immunsystems und könnten entscheidend zum Toleranzbruch beitragen [23].

## Antigenpräsentation

Die Präsentation von Antigenen erfolgt primär durch dendritische Zellen, denn nur sie können naive T‑Zellen aktivieren. Im weiteren Verlauf der Immunreaktion gelingt sie auch sehr effizient durch antigenspezifische B‑Zellen, da diese auch geringste Mengen ihres spezifischen Antigens mithilfe ihrer B‑Zell-Rezeptoren aufnehmen und präsentieren können. Die wirkungsvolle Antigenpräsentation hängt einerseits von der individuellen Ausstattung an HLA-Molekülen, andererseits vom Aktivierungszustand der antigenpräsentierenden Zellen ab. Dabei ist der SLE mit HLA-DR3 und HLA-DR15, vermutlich auch HLA-DR9 assoziiert [7].

Wesentliche Faktoren für die Aktivierung antigenpräsentierender Zellen stellen die Interferone dar, die insbesondere (konventionelle) dendritische Zellen aktivieren und die Antigenpräsentation verbessern, was gut zu ihrer physiologischen Rolle in der Virusabwehr passt.

## T-Zellen

Dass T‑Zellen beim SLE eine Rolle spielen, ist schon durch die Tatsachen der deutlichen HLA-Assoziation des SLE und der T‑Zell-Hilfe für die Autoantikörperproduktion gegeben. Diese T‑Zell-Hilfe lässt sich auch in vitro nachbilden [24, 25]. Autoreaktive T‑Zellen können das Autoantigen dabei im MHC-Kontext entweder durch dendritische Zellen oder durch gegen das gleiche Antigen gerichtete B‑Zellen präsentiert bekommen, weniger effizient auch durch Makrophagen. Für die Aktivierung der B‑Zellen spielt dann aus heutiger Sicht die Kostimulation von CD40-Ligand (CD40L) auf T‑Zellen zu CD40 auf B‑Zellen eine wichtige Rolle. Die Blockade dieser Achse beim SLE wird derzeit mittels zweier medikamentöser Ansätze, Dapirolizumab pegol (gegen CD40L) und Iscalimab (gegen CD40), getestet. Für Dapirolizumab sind Phase-II-Ergebnisse publiziert [26].

Unterstützt wird die Bedeutung der Lupus-T-Zellen durch Daten zur niedrig dosierten IL-2-Gabe bei SLE [27, 28]. Beim SLE herrscht auf Basis veränderter Signaltransduktionsmechanismen ein relativer IL-2-Mangel vor [29]. Während höhere IL-2-Dosen zur Aktivierung inflammatorischer Effektor-T-Zell-Antworten beitragen könnten, führt die Zufuhr in niedriger Dosis zum selektiven Überlebensvorteil von regulatorischen T‑Zellen (Treg), die hohe Konzentrationen des hochaffinen IL-2-Rezeptors an ihrer Oberfläche tragen [30]. Die beobachtete klinische Besserung in diesen Studien spricht auch für eine relevante Rolle der Treg bzw. eines Funktionsdefizits oder Mangels von Treg beim SLE.

## B-Zellen

Obwohl bestimmte Mutationen [19] auf diesem Weg zum SLE führen (Tab. 2), sind mit Sicherheit nicht alle Faktoren bekannt, die dazu führen, dass im Rahmen des SLE verschiedenste autoimmune B‑Zellen überleben und teilweise zu langlebigen Plasmazellen werden. Solche Mechanismen einer unvollständigen negativen Selektion, also des Überlebens autoimmuner B‑Zellen, müssen aber vorhanden sein, weil die SLE-Autoantikörper nicht monoklonal, sondern zumindest oligoklonal, wenn nicht gar polyklonal sind. Es werden also immer mehrere autoimmune B‑Zellen aktiviert.

Ein diesbezüglich plausibler Umweltfaktor ist die Infektion mit Epstein-Barr-Virus (EBV) [31]. EBV führt zu einer drastischen, polyklonalen B‑Zell-Aktivierung. Während fast alle jungen Erwachsenen diese Infektion bereits hinter sich haben, ist das bei Kindern und Jugendlichen nicht der Fall. Bei Kindern und Jugendlichen mit SLE wurde hingegen in 100 % eine stattgehabte EBV-Infektion nachgewiesen [32].

Ein endogener Faktor ist das B‑Zell-Zytokin B‑Lymphozyten-Stimulator (BLyS) oder B‑Zellen-aktivierender Faktor (BAFF). BLyS/BAFF-Überproduktion ist ein genetischer Risikofaktor für den SLE [33], und BLyS/BAFF ist bei Patienten mit aktivem SLE deutlich erhöht [34, 35]. Das Zytokin bindet v. a. 2 unterschiedliche Rezeptoren (BAFF-Rezeptor, TACI – „transmembrane activator‑1 and calcium modulator and cyclophilin ligand-interactor“) auf B‑Lymphozyten und zum Teil Plasmazellen und kann zu einer unvollständigen negativen Selektion autoimmuner B‑Zellen führen [36].

## Plasmablasten und langlebige Plasmazellen

Während B‑Zellen wesentliche Funktionen als antigenpräsentierende Zellen und für das Immungedächtnis („memory B cells“) haben, müssen sie für die Antikörperproduktion in Richtung Plasmazellen ausdifferenzieren [37]. Zunächst entstehen Plasmablasten, die noch proliferieren und dann zu nicht mehr vermehrungsfähigen Plasmazellen differenzieren. Während die Mehrzahl der Plasmazellen nur wenige Tage überlebt, können bestimmte Plasmazellen zu sog. langlebigen Plasmazellen werden, vorausgesetzt, sie finden eine Plasmazellnische, aus der sie ggf. eine andere Plasmazelle verdrängen. Sowohl rezent differenzierte Plasmablasten als auch langlebige Plasmazellen spielen beim SLE eine Rolle [38].

In den mutmaßlichen Plasmazellnischen, die wohl überwiegend aus mesenchymalen Zellen bestehen, erhalten Plasmazellen Überlebenssignale in Form von Zytokinen (z. B. APRIL) und direkten Zell-Zell-Kontakten, sodass sie dort als langlebige Plasmazellen über Jahrzehnte überleben und ihren jeweiligen monoklonalen Antikörper produzieren können [39]. Von langlebigen Plasmazellen sezernierte neutralisierende Antikörper sind verantwortlich für den lebenslangen Schutz vor Kinderkrankheiten wie Mumps und Masern nach einer durchgemachten Infektion oder Impfung. Langlebige Plasmazellen finden sich besonders im Knochenmark, in der Milz, aber auch in chronisch entzündeten Organen, z. B. in der Niere bei Lupusnephritis [40, 41]. Langlebige Plasmazellen, die pathogene Antikörper produzieren, können jedoch auch die Ursache therapierefraktärer Autoimmunkrankheiten darstellen, da diese Plasmazellen durch konventionelle Therapien wie hoch dosierte Glukokortikoide, Cyclophosphamid, Mycophenolat oder Rituximab praktisch nicht angreifbar sind. Einige experimentelle Therapien, die jedoch zumindest für den SLE nicht zugelassen sind, können auch langlebige Plasmazellen zumindest teilweise eliminieren. Hierzu gehören die Hochdosischemotherapie mit autologer Stammzelltransplantation kombiniert mit Anti-Thymozyten-Globulin, die durch ein „immunologisches Reset“ sogar medikamentenfreie Remissionen erzielen kann, die für die Myelomtherapie zugelassenen Proteasominhibitoren (z. B. Bortezomib) und Anti-CD38-Antikörper (z. B. Daratumumab) sowie das neben BAFF auch APRIL neutralisierende TACI-Ig-Fusionsprotein Atacicept.

## Direkte Autoantikörpereffekte

Ein Teil der SLE-Autoantikörper bindet direkt an zugängliche Antigene. Am einfachsten ist das anhand der Autoantikörper gegen Blutkörperchen erklärbar [42, 43]. Durch den Coombs-Test direkt nachweisbar sind die Autoantikörper gegen Erythrozytenzellmembranen im Rahmen der autoimmunhämolytischen Anämie. Mit Antikörpern beladene Erythrozyten werden in der Folge auch mit Komplementspaltprodukten bedeckt. Das führt v. a. über Komplement- und Fc-Rezeptoren zur Phagozytose in Milz und Leber, andererseits über den „membrane attack complex“ (MAC), der als Pore in der sonst dichten Zellmembran wirkt, auch zur direkten Lyse von Erythrozyten. Auch wenn Autoantikörper gegen Thrombozyten, Granulozyten und Lymphozyten beim SLE in der Regel nicht direkt nachgewiesen werden, sind die Vorgänge bei diesen Zytopenien meist analog derer bei der autoimmunhämolytischen Anämie. (Möglicherweise können auch autoreaktive zytotoxische T‑Zellen die Hämatopoese beeinträchtigen). Alternativ kann es aber auch bereits zu Effekten im Rahmen der Bildung im Knochenmark kommen, was sich dann in Form verminderter Zellreihen in der Knochenmarkstanze zeigt. Ähnliche, wenn auch im ZNS lokalisierte Effekte, ergeben sich durch Autoantikörper gegen Aquaporin 4, die zum Untergang von Neuronen führen können [44, 45].

Ebenfalls gut erklärbar sind die Effekte von Antiphospholipidantikörpern [46, 47]. Kommt es zu einer (gewissen) Aktivierung von Thrombozyten oder Endothelzellen, werden Cardiolipin und das daran gebundene Beta-2-Glykoprotein‑I für die Autoantikörper zugänglich, was zu einer kompletten Aktivierung mit Bildung venöser und/oder arterieller Thromben führt. Dadurch ist auch die häufige, meist leichte Thrombopenie beim Antiphospholipidsyndrom erklärbar.

Schwieriger sind direkte, funktionelle ZNS-Effekte zu erklären, die unter anderem infolge von Antikörpern gegen ribosomale P‑Proteine auftreten können [48]. Da hier keine Apoptose von Nervenzellen nachweisbar ist, verursachen diese Antikörper offenbar reversible Fehlfunktionen. Sie werden außerhalb des ZNS produziert; das medikamentöse „Schließen“ der offenen Blut-Hirn-Schranke durch sehr hoch dosierte Glukokortikoide und deren breit antientzündlichen Wirkungen führen zu einer umgehenden Besserung psychotischer Symptome.

## Immunkomplexe

Die große Gruppe der sehr unterschiedlichen entzündlichen Manifestationen des SLE werden hingegen v. a. durch Immunkomplexe ausgelöst [5], die häufig an (negativ geladenen) Basalmembranen gebildet werden, aber durchaus auch in der Zirkulation vorkommen. In diesem Fall handelt es sich nahezu ausschließlich um IgG-Immunkomplexe, die auf der Y‑Form der Immunglobuline mit 2 Bindungsstellen beruhen. Art des Immunkomplexes und Ort der Ablagerung bedingen dabei die Organmanifestation. So sind Komplexe aus doppelsträngiger DNA bzw. Nukleosomen und Anti-dsDNA-Antikörpern auf den glomerulären Basalmembranen die Auslöser der proliferativen (Klasse III und IV) Lupusnephritiden [49]. Ro-anti-Ro-Antikörper-Komplexe im Bereich der Basalmembran in der dermoepidermalen Junktionszone der Haut liegen den subakut kutanen (SCLE-)Manifestationen und dem neonatalen Lupus zugrunde [50, 51].

Immunkomplexe können an ihren Fc-Teilen (dem Stiel des Ypsilons) v. a. von Monozyten und Makrophagen über deren Fc-Rezeptoren gebunden werden. Fc-Rezeptor-Knockout-Mäuse ohne proinflammatorische Fcγ-Rezeptoren sind vor einer Lupusnephritis geschützt [52]. Immunkomplexe führen aber auch zur Komplementaktivierung über den klassischen Weg [53]. C1q bindet an die Komplementbindungsstelle des Fc-Teils und der C1qrs-Komplex spaltet und aktiviert C4 (zu C4b und C4a) und dann C2 im Komplex C4b2 zu C4b2a. In der Folge werden C3 und C5 gespalten; auf C5b baut sich als C5b6789 der oben bei den direkten Antikörpereffekten erwähnte MAC auf. Entscheidend für die Organschädigung dürfte die Freisetzung von C3a und C5a sein, die chemotaktisch wirken und das Einströmen von Entzündungszellen veranlassen.

## Zytokine

Sowohl Monozyten/Makrophagen als auch plasmazytoide dendritische Zellen (pDC) reagieren mit der Produktion von Zytokinen, wenn sie Immunkomplexe erkennen. Makrophagen produzieren dabei einerseits proinflammatorische Zytokine wie Tumornekrosefaktor (TNF) [54] oder IL-6 [55], andererseits aber BLyS/BAFF [56]. Die proinflammatorischen Zytokine tragen auch beim SLE zur Gewebeentzündung bei, wie Ergebnisse offener Studien mit TNF-Blockern [57, 58] und dem IL-6-Rezeptor-Blocker Tocilizumab [59] zeigen (Tab. 3).

**Table 3:** 

Ziel	Ansatz/Substanz	Evidenz	Bei welcher Organbeteiligung
B‑Zellen	Obinutuzumab	Phase II	Lupusnephritis
Regulatorische T‑Zellen	IL‑2 niedrig dosiert	Phase II	Lupusnephritis, nichtrenaler SLE
T‑B-Kostimulation CD40L	Dapirolizumab pegol	Phase II	Nichtrenaler SLE
Plasmablasten	Cyclophosphamid^a^	Phase III	Lupusnephritis
Langlebige Plasmazellen	ASCT	Offen	Lupusnephritis, nichtrenaler SLE
	Bortezomib	Offen	Lupusnephritis, nichtrenaler SLE
Autoantikörper	Immunadsorption	Offen	Lupusnephritis, nichtrenaler SLE
TNF	Infliximab	Offen	Lupusnephritis
	Etanercept	Offen	Nichtrenaler SLE
BLyS/BAFF	Belimumab	Phase III	Lupusnephritis, nichtrenaler SLE
IL‑6	Tocilizumab	Offen	Lupusarthritis
	Baricitinib	Phase II	Nichtrenaler SLE
Typ-I-Interferone	Anifrolumab	Phase III	Nichtrenaler SLE
	Anifrolumab	Phase II	Lupusnephritis
	Baricitinib	Phase II	Nichtrenaler SLE

^a^Cyclophosphamid wirkt aber unter anderem auch auf T- und B‑Lymphozyten

Hingegen ist BLyS/BAFF kein proinflammatorisches Effektorzytokin, sondern bewirkt eine positive Feedbackschleife. Immunkomplexe führen zur Bildung von BLyS/BAFF; BLyS/BAFF fördert das Überleben auch autoimmuner B‑Zellen und erhöht so die Last an Autoantikörpern und Immunkomplexen. Dieser Mechanismus ist im Fall systemischer Virusinfektionen, bei denen es zur Bildung von Immunkomplexen kommt, Teil der physiologischen Immunreaktion, führt aber beim SLE zu einem Circulus vitiosus. Da Rezeptoren für BLyS/BAFF nur auf B‑Zellen vorhanden sind, erhöhen sich die BLyS/BAFF-Spiegel bei B‑Zell-Mangel, insbesondere nach B‑Zell-Depletion [60].

Die direkte Blockade von BLyS führt zu einem relativ raschen Anstieg niedriger Komplement-C3- und -C4-Spiegel [61], vermutlich durch Rückgang von Immunkomplexen. Autoantikörper fallen über die Zeit leicht ab, die Gesamtimmunglobuline werden aber in aller Regel nicht relevant vermindert [62]. Hingegen führt Atacicept zu einem deutlichen Abfall von IgG [63]. Atacicept besteht aus dem Fc-Teil des humanen IgG und der extrazellulären Domäne von TACI, einem Rezeptormolekül, das sowohl BLyS als auch APRIL bindet. Das TACI-IgG-Hybridmolekül blockiert somit zusätzlich zu BLyS das Schwesterzytokin APRIL („a proliferation-inducing ligand“) und beeinträchtigt so auch das Plasmazellüberleben.

Im Gegensatz zu den Makrophagen produzieren pDC als Antwort auf (DNA- oder RNA-haltige) Immunkomplexe Typ-I-Interferone, insbesondere Interferon‑α (IFNα) [64]. IFNα hat neben antiviralen und Apoptose-fördernden Eigenschaften einen erheblichen Einfluss auf die Ausreifung und Aktivierung konventioneller dendritischer Zellen [65] und fördert seinerseits wieder die BLyS-Sekretion [56].

Der stärkere Effekt von Anifrolumab [66], das den gemeinsamen Rezeptor aller Typ-I-Interferone (neben Interferon-α (IFNα) auch IFNβ, IFNε, IFNκ und IFNω) blockiert, im Vergleich zum Anti-IFNα-Antikörper Sifalimumab [67] kann ein Hinweis für eine Rolle anderer Typ-I-Interferone sein. In der Lupushaut spielt jedenfalls epidermal produziertes IFNκ eine induzierende Rolle [68]. Dass Typ-I-Interferone beim SLE eine Rolle spielen, ergibt sich auch aus einer Reihe von Beschreibungen SLE-assoziierter genetischer Veränderungen, die zur vermehrten Interferonsekretion führen (Tab. 2; [19]), und aus der lange bekannten Tatsache der Auslösbarkeit eines SLE durch die therapeutische Gabe von Typ-I-Interferon z. B. bei Virushepatitiden [69].

## Schlussfolgerung

Auch wenn wir die Pathophysiologie des SLE bei Weitem nicht komplett verstehen, haben die Forschungsergebnisse der letzten Jahrzehnte und die neuen Therapien eine Menge neuer Puzzleteile beigesteuert, die sich zunehmend zu einem Bild zusammenfügen. Das SLE-Risiko steigt mit Faktoren, die zur B‑Zell-Aktivierung oder zum Überleben autoreaktiver B‑Zellen beitragen, mit Störungen in der Abräumung apoptotischen Zellmaterials, das viele SLE-assoziierte Autoantigene enthält, und mit chronischem Interferoneinfluss als „Virus-Gefahrensignal“. Pathogene Autoantikörper werden sowohl von kurzlebigen Plasmablasten als auch von langlebigen Plasmazellen produziert, wobei Letztere weitgehend resistent gegen konventionelle Immunsuppressiva sind. Die Autoantikörper wirken zum Teil direkt auf Zellen und können in Form von Immunkomplexen und mithilfe von Komplement Makrophagen aktivieren, die über proinflammatorische Zytokine die Entzündungsreaktion befeuern und über das B‑Zell-Zytokin BLyS/BAFF sowie Interferone in einer positiven Rückkopplungsschleife zum Überleben autoreaktiver Lymphozyten und zur Bildung weiterer Autoantikörper beitragen. Neue Therapien ergeben dort gezielte Ansätze.
